# Authenticity of Honey: Characterization, Bioactivities and Sensorial Properties Series II

**DOI:** 10.3390/foods13132079

**Published:** 2024-07-01

**Authors:** Olga Escuredo, M. Carmen Seijo

**Affiliations:** Department of Vegetal Biology and Soil Science, Faculty of Science, University of Vigo, As Lagoas, 32004 Ourense, Spain; mcoello@uvigo.es

Honey is a food product globally appreciated by consumers due to its extremely reduced processing requirements and its nutritional properties. The characteristics of honey are defined based on its sugar profile, mineral elements, quality parameters, bioactive compounds, antioxidants, antimicrobial properties, sensorial attributes, and palynological profile. The particularities of honey composition are attributed to its botanical and geographical origin. However, in some honey types, the identification of these physicochemical and sensory characteristics related to botanical origin remains scarce. Currently, the methods for confirming honey floral origins are limited and some require improvements. The lack of specific legislation for each type of honey based on precise results extracted from palynological methods, chromatographic or spectroscopic techniques, or sensory analyses highlights the need for the scientific community to continue working on the transfer to the beekeeping sector. Therefore, there is demand from the beekeeping sector and consumers to authenticate honey linked to specific botanical origins. The success of combining physicochemical, palynological, and sensory data with chemometric treatment using various clustering and classification algorithms to discriminate the origin of honey has been revealed in some of the documents published. For this reason, knowledge regarding the physicochemical, sensorial, and palynological characteristics of honey continues to be in high demand.

In this Special Issue, we publish research papers on the authenticity, physicochemical characterization by honey type, sensory characteristics, and crystallization states related to consumer acceptance and the botanical origin of the honey. The identification of honey origin based on physicochemical and sensory profiles or specific chemical markers that guarantee the authenticity of honey is a useful tool for quality assurance and traceability. The papers selected for this Special Issue were subject to a rigorous peer review procedure, with the aim of the rapid and wide dissemination of research results, developments, and applications in the beekeeping sector. We hope that research such as that included in this Special Issue can provide rigorous scientific information on certain honey types and unknown honeys collected worldwide.

The objectives of the published papers were as follows. Bouddine et al. [1] characterized organic honeys produced in the Middle Atlas region of Morocco based on physicochemical parameters, ascorbic acid, and phenolic and flavonoid compounds, as well as the antioxidant properties, pollen spectra, and sugar profile. A palynological analysis revealed the dominance of pollens such as *Ziziphus lotus*, Rhamnaceae, *Sinapis arvensis*, Fabaceae, Apiaceae, Lamiaceae, *Rosmarinus officinalis*, and *Thymus vulgaris* in the honeys analyzed. High antioxidant activity and phytochemical constituents in some honeys were found. The pollen profile and its relationship with the physicochemical characteristics in Moroccan honeys are little studied. Therefore, the results of the study provide valuable information for beekeepers, and these honeys can be used as bioactive ingredients in the nutraceutical and food industries for multiple purposes. The authors of the research highlight the importance of the results obtained in increasing the commercial value of honey and note that it could have a significant economic impact for the rural population of Morocco.

Chuach et al. [2] investigated the antioxidant activities of monofloral honeys from acacia, agarwood, coconut, dwarf mountain pine, creeper, rubber, and star fruit produced by stingless bees (*Heterotrigona itama*). This work provides the identification of potential antioxidant markers in stingless bee honeys associated with the botanical origin of the nectar. A non-targeted liquid chromatography–mass spectrometry (LC-MS) metabolomic approach was used to identify antioxidant compounds that could explain specific compounds attributed to botanical origin. The antioxidants identified were mainly alkaloids and flavonoids. In the case of acacia honey, the researchers discovered compounds derived from flavonoids as possible key markers of this honey type. A holistic approach associating metabolite data with supervised chemometric analysis was useful for the identification of specific compound markers related to a specific botanical origin. Compositional differences between the analyzed monofloral honeys were determined using a direct mass spectrometry approach coupled with chemometrics. These results are valuable for authenticating the botanical origin of these honeys. However, it is necessary to extend the analysis of the composition of antioxidant compounds to a larger number of samples to verify the compounds selected as reliable markers for quality control.

The paper presented by Labsvards et al. [3] included the multi-element profile using ICP-MS of 83 polyfloral honeys from Latvia and a comparison with honey samples from other geographical origins. Element profiling, as a method for evaluating the floral origins of the honey, was possible. Among the objectives of the study was to provide chemical information to Latvian beekeepers on honeys from this geographical area with specific floral sources, complementing the authentication of the honey. A melissopalynology analysis identified the botanical source of honeys and was related to each mineral element. A multi-method approach was successful in identifying heather honey by evaluating the concentrations of Ca, Fe, Rb, Cs, and Ba. For buckwheat honey, the concentration of Cu and for willow or linden honey, the concentration of Sn can be valuable secondary indicators. Multi-element profiles of proteins of buckwheat, clover, and polyfloral honeys were investigated to clarify whether most of the elements were protein-bound or not. The results indicated that Ca, K, Mg, Mn, Na, and Sr were mainly found in non-protein-bound forms; thus, they are possibly also bioavailable in honey, but Al, Cu, Ni, and Zn were found in the form of large chemical structures.

The main purpose of the study presented by Escuredo et al. [4] was to analyze unifloral and multifloral honeys (362 samples) produced in the northwest of Spain using chemometric techniques. Specifically, principal component analysis and discriminant analysis were successfully applied to study the relationships between the main physicochemical variables (moisture, pH, electrical conductivity, diastase content, phenols, flavonoids, and color) and dominant pollen variables extracted from the melissopalynological analysis. Linear discriminant analysis correctly classified 89.8% of the samples according to their botanical origin. Unifloral honeys from blackberry, eucalyptus, and heather were correctly grouped, while five chestnut honeys and fourteen samples of honeydew honeys were misclassified. The physicochemical variables with higher importance in the differentiation of honeys according to botanical origin were electrical conductivity, total phenol, and flavonoid contents, together with the dominant pollens (*Eucalyptus*, *Erica*, *Rubus*, and *Castanea sativa*). The results showed the potential of multivariate statistics in the characterization of honey of the same geographical origin, a challenge for many researchers in the study of honeys from other geographic origins. Honeydew and chestnut honeys produced in Galicia (NW Spain) have similar physicochemical characteristics, and sometimes honeys have a similar pollen profile. Therefore, multivariate techniques applied with a wide list of data that include chemical compounds, physicochemical parameters, and pollen contribute to the differentiation of some types of honey satisfactorily. At the same time, in this research, the antioxidant properties and polyphenol content of a high number of unifloral samples were presented, contributing to a good characterization of these honeys covered by Protected Geographical Indication (PGI) *Miel de Galicia*.

Meixner et al. [5] investigated the parameters that influence the honey agitation process and can favor better dissolution of the crystals to obtain a creamier honey for marketing. Sometimes, consumers associate crystallized honey with improper handling or poor quality, having a negative impact on marketing. The crystallization behavior of honey depends on its composition (mainly the glucose to fructose ratio) and botanical origin. Honey stirring is a complex process in which several factors intervene in the creaminess of this beekeeping product. This study focused on the measurement of five stirring parameters, including the type of stirring device, honey pretreatment, stirring temperature (14 °C to room temperature), stirring range (1 to 24 times) and the stirring time (from 1 to 15 min). For this, spring flower honeys collected in Saxony-Anhalt (Germany) were analyzed. The results showed the importance of temperature during honey handling. If the optimal temperature of 14 °C cannot be maintained, some beekeeping practices are recommended, such as sifting the honey with a mesh before stirring it, using a screw stirrer, and stirring several times a day for a good dissolution of the crystals present in the honey.

Piana et al. [6] analyzed the sensory properties, consumer perception, and acceptance of two different monofloral honeys (citrus honey and rape honey), both with different aromatic properties and different levels of acceptability by consumers. For each monofloral honey, three different physical states were considered (liquid, creamy, and crystallized). Physicochemical characteristics and a dynamic sensory analysis, consumer test, and CATA test on the three honey textures were conducted. In addition, it was taken into account how processing methods influence texture properties. In general, the liquid honey samples were sweeter, but less aromatic. Rheological analysis of citrus and rape honeys showed differences in the three crystallization states. Consumer testing validated the panel data and confirmed consumers’ increased appreciation for liquid and creamy honey. Therefore, these differences were not only related to botanical origin, but also to levels of granulation. The CATA data related to visual/texture properties were able to identify the attributes that better describe the samples. The ANOVA factorial analysis showed that the floral and sweetness attributes had a moderately significant effect on the crystallization parameter, and it was mainly related to the botanical origin (72.97% of the variance). Notably, the two honeys chosen for the study represent two different levels of acceptability related to different aromatic properties. Consumers widely appreciate citrus honey characterized by floral, fruity, and sweet notes and a more homogeneous crystallization. However, the particularity of rape honey lies in its texture and complexity of flavor, which could be interesting for promotion by beekeepers and distributors. Therefore, the controlled crystallization of honey is a valuable instrument to influence consumer acceptance, with botanical origin playing an important role with respect to sensory attributes.

In summary, the six papers published in this Special Issue made a significant contribution and helped to deepen the understanding of the topic of “Authenticity of Honey: Characterization, Bioactivities and Sensorial Properties Series II”. These documents are an example of the efforts of researchers in this field of beekeeping, collaborating closely with the local beekeeper in the recognition of high-quality products, and in the acceptance of this beekeeping product at a national and international level. Simultaneously, the recognized healthy and therapeutic properties will have a positive impact on the marketing of honey. We thank the authors for sharing their valuable contributions in this second Special Issue. Together with them, we emphasize the need to carry out more research with the purpose of providing more knowledge of the characteristics of honey from different types of view and for different purposes in the food or pharmaceutical industry.

## Data Availability

Not applicable.
